# Supplementary material for: Resolving the full spectrum of human genome variation using Linked-Reads
Source: Genome Res. 2019 Apr;29(4):635–45. doi: 10.1101/gr.234443.118 (PMC6442396; doi:10.1101/gr.234443.118)
Supplement: Supplemental Material [file supp_gr.234443.118_Supplemental_longranger-2.2.zip › longranger-2.2.tar.bz2!/lr2.2-public/lib/stan/cmdstan/stan/doc/api/html/agrad_2rev_2matrix_2trace__gen__quad__form_8hpp_source.html]

Stan: src/stan/agrad/rev/matrix/trace\_gen\_quad\_form.hpp Source File


|  |  |
| --- | --- |
|  | Stan  2.5.0  probability, sampling & optimization |


- Main Page
- Related Pages
- Namespaces
- Classes
- Files

- File List
- File Members

All Classes Namespaces Files Functions Variables Typedefs Enumerations Enumerator Friends Macros Pages

- src
- stan
- agrad
- rev
- matrix

trace\_gen\_quad\_form.hpp

Go to the documentation of this file.

1 #ifndef STAN\_\_AGRAD\_\_REV\_\_MATRIX\_\_TRACE\_GEN\_QUAD\_FORM\_HPP

2 #define STAN\_\_AGRAD\_\_REV\_\_MATRIX\_\_TRACE\_GEN\_QUAD\_FORM\_HPP

3

4 #include <boost/utility/enable\_if.hpp>

5 #include <boost/type\_traits.hpp>

6 #include <stan/math/matrix/Eigen.hpp>

7 #include <stan/math/matrix/typedefs.hpp>

8 #include <stan/agrad/rev/matrix/value\_of.hpp>

9 #include <stan/agrad/rev/var.hpp>

10 #include <stan/agrad/rev/vari.hpp>

11 #include <stan/agrad/rev/matrix/typedefs.hpp>

12 #include <stan/math/matrix/trace\_gen\_quad\_form.hpp>

13 #include <stan/math/error\_handling/matrix/check\_multiplicable.hpp>

14 #include <stan/math/error\_handling/matrix/check\_square.hpp>

15

16 namespace stan {

17  namespace agrad {

18  namespace {

19  template<typename TD,int RD,int CD,

20  typename TA,int RA,int CA,

21  typename TB,int RB,int CB>

22  class trace\_gen\_quad\_form\_vari\_alloc : public chainable\_alloc {

23  public:

24  trace\_gen\_quad\_form\_vari\_alloc(const Eigen::Matrix<TD,RD,CD> &D,

25  const Eigen::Matrix<TA,RA,CA> &A,

26  const Eigen::Matrix<TB,RB,CB> &B)

27  : D\_(D), A\_(A), B\_(B)

28  { }

29

30  double compute() {

31  return stan::math::trace\_gen\_quad\_form(value\_of(D\_),

32  value\_of(A\_),

33  value\_of(B\_));

34  }

35

36  Eigen::Matrix<TD,RD,CD> D\_;

37  Eigen::Matrix<TA,RA,CA> A\_;

38  Eigen::Matrix<TB,RB,CB> B\_;

39  };

40

41  template<typename TD,int RD,int CD,

42  typename TA,int RA,int CA,

43  typename TB,int RB,int CB>

44  class trace\_gen\_quad\_form\_vari : public vari {

45  protected:

46  static inline void computeAdjoints(const double &adj,

47  const Eigen::Matrix<double,RD,CD> &D,

48  const Eigen::Matrix<double,RA,CA> &A,

49  const Eigen::Matrix<double,RB,CB> &B,

50  Eigen::Matrix<var,RD,CD> \*varD,

51  Eigen::Matrix<var,RA,CA> \*varA,

52  Eigen::Matrix<var,RB,CB> \*varB)

53  {

54  Eigen::Matrix<double,CA,CB> AtB;

55  Eigen::Matrix<double,RA,CB> BD;

56  if (varB || varA)

57  BD.noalias() = B\*D;

58  if (varB || varD)

59  AtB.noalias() = A.transpose()\*B;

60

61  if (varB) {

62  Eigen::Matrix<double,RB,CB> adjB(adj\*(A\*BD + AtB\*D.transpose()));

63  int i,j;

64  for (j = 0; j < B.cols(); j++)

65  for (i = 0; i < B.rows(); i++)

66  (\*varB)(i,j).vi\_->adj\_ += adjB(i,j);

67  }

68  if (varA) {

69  Eigen::Matrix<double,RA,CA> adjA(adj\*(B\*BD.transpose()));

70  int i,j;

71  for (j = 0; j < A.cols(); j++)

72  for (i = 0; i < A.rows(); i++)

73  (\*varA)(i,j).vi\_->adj\_ += adjA(i,j);

74  }

75  if (varD) {

76  Eigen::Matrix<double,RD,CD> adjD(adj\*(B.transpose()\*AtB));

77  int i,j;

78  for (j = 0; j < D.cols(); j++)

79  for (i = 0; i < D.rows(); i++)

80  (\*varD)(i,j).vi\_->adj\_ += adjD(i,j);

81  }

82  }

83

84

85  public:

86  trace\_gen\_quad\_form\_vari(trace\_gen\_quad\_form\_vari\_alloc<TD,RD,CD,TA,RA,CA,TB,RB,CB> \*impl)

87  : vari(impl->compute()), \_impl(impl) { }

88

89  virtual void chain() {

90  computeAdjoints(adj\_,

91  value\_of(\_impl->D\_),

92  value\_of(\_impl->A\_),

93  value\_of(\_impl->B\_),

94  (Eigen::Matrix<var,RD,CD>\*)(boost::is\_same<TD,var>::value?(&\_impl->D\_):NULL),

95  (Eigen::Matrix<var,RA,CA>\*)(boost::is\_same<TA,var>::value?(&\_impl->A\_):NULL),

96  (Eigen::Matrix<var,RB,CB>\*)(boost::is\_same<TB,var>::value?(&\_impl->B\_):NULL));

97  }

98

99  trace\_gen\_quad\_form\_vari\_alloc<TD,RD,CD,TA,RA,CA,TB,RB,CB> \*\_impl;

100  };

101  }

102

103  template<typename TD,int RD,int CD,

104  typename TA,int RA,int CA,

105  typename TB,int RB,int CB>

106  inline typename

107  boost::enable\_if\_c< boost::is\_same<TD,var>::value ||

108  boost::is\_same<TA,var>::value ||

109  boost::is\_same<TB,var>::value,

110  var >::type

111  trace\_gen\_quad\_form(const Eigen::Matrix<TD,RD,CD> &D,

112  const Eigen::Matrix<TA,RA,CA> &A,

113  const Eigen::Matrix<TB,RB,CB> &B)

114  {

115  stan::math::check\_square("trace\_gen\_quad\_form(%1%)",A,"A",(double\*)0);

116  stan::math::check\_square("trace\_gen\_quad\_form(%1%)",D,"D",(double\*)0);

117  stan::math::check\_multiplicable("trace\_gen\_quad\_form(%1%)",A,"A",

118  B,"B",(double\*)0);

119  stan::math::check\_multiplicable("trace\_gen\_quad\_form(%1%)",B,"B",

120  D,"D",(double\*)0);

121

122  trace\_gen\_quad\_form\_vari\_alloc<TD,RD,CD,TA,RA,CA,TB,RB,CB> \*baseVari = new trace\_gen\_quad\_form\_vari\_alloc<TD,RD,CD,TA,RA,CA,TB,RB,CB>(D,A,B);

123

124  return var(new trace\_gen\_quad\_form\_vari<TD,RD,CD,TA,RA,CA,TB,RB,CB>(baseVari));

125  }

126  }

127 }

128

129 #endif

check\_multiplicable.hpp

vari.hpp

typedefs.hpp

typedefs.hpp

stan::agrad::trace\_gen\_quad\_form

fvar< T > trace\_gen\_quad\_form(const Eigen::Matrix< fvar< T >, RD, CD > &D, const Eigen::Matrix< fvar< T >, RA, CA > &A, const Eigen::Matrix< fvar< T >, RB, CB > &B)

**Definition:** trace\_gen\_quad\_form.hpp:15

stan::agrad::value\_of

T value\_of(const fvar< T > &v)

Return the value of the specified variable.

**Definition:** value\_of.hpp:16

stan::math::check\_multiplicable

bool check\_multiplicable(const char \*function, const T1 &y1, const char \*name1, const T2 &y2, const char \*name2, T\_result \*result)

**Definition:** check\_multiplicable.hpp:15

value\_of.hpp

\_impl

trace\_gen\_quad\_form\_vari\_alloc< TD, RD, CD, TA, RA, CA, TB, RB, CB > \* \_impl

**Definition:** trace\_gen\_quad\_form.hpp:99

B\_

Eigen::Matrix< TB, RB, CB > B\_

**Definition:** trace\_gen\_quad\_form.hpp:38

D\_

Eigen::Matrix< TD, RD, CD > D\_

**Definition:** trace\_gen\_quad\_form.hpp:36

var.hpp

Eigen.hpp

A\_

Eigen::Matrix< TA, RA, CA > A\_

**Definition:** trace\_gen\_quad\_form.hpp:37

stan::agrad::var

Independent (input) and dependent (output) variables for gradients.

**Definition:** var.hpp:27

trace\_gen\_quad\_form.hpp

stan::math::trace\_gen\_quad\_form

double trace\_gen\_quad\_form(const Eigen::Matrix< double, RD, CD > &D, const Eigen::Matrix< double, RA, CA > &A, const Eigen::Matrix< double, RB, CB > &B)

Compute trace(D B^T A B).

**Definition:** trace\_gen\_quad\_form.hpp:17

stan::math::check\_square

bool check\_square(const char \*function, const Eigen::Matrix< T\_y, Eigen::Dynamic, Eigen::Dynamic > &y, const char \*name, T\_result \*result)

Return true if the specified matrix is square.

**Definition:** check\_square.hpp:24

check\_square.hpp


---

[ Stan Home Page ]

*© 2011–2014,
Stan Development Team.*
